# Quality of Life Among Saudi Mothers of Children with Autism Spectrum Disorder: Associations with Caregiver Burden, Psychological Distress, Financial Experiences, and Post-Diagnosis Service Pathway Clarity

**DOI:** 10.3390/healthcare14172847

**Published:** 2026-09-04

**Authors:** Nisreen N. Al Awaji, Anfal A. Aldursuni, Fay M. Almutairi, Asma M. Alanazi, Retaj A. Alanazi, Bayan A. Alhurayyis, Reemaz A. Alwajee, Atheer Alghamdi, Shaden Alabdulkarim

**Affiliations:** 1 Speech and Swallowing Pathology Program, Department of Health Communication Sciences, College of Health and Rehabilitation Sciences, Princess Nourah bint Abdulrahman University, Riyadh 84428, Saudi Arabia; 2 Rehabilitation Department, King Abdullah Bin Abdulaziz University Hospital, Riyadh 11564, Saudi Arabiasaalabdulkarim@kaauh.edu.sa (S.A.)

**Keywords:** autism spectrum disorder, quality of life, mothers, caregiver burden, service pathways, financial burden, Saudi Arabia

## Abstract

**Highlights:**

**What are the main findings?**

- Caregiver burden was the factor most strongly associated with lower maternal quality of life among Saudi mothers of children with ASD.
- Higher household income and clearer post-diagnosis service pathways were each associated with better maternal quality of life in the multivariable model.
- Caregiver and service-related variables together explained 59.3% of the variation in maternal quality of life.
- Mothers’ brief comments highlighted therapy affordability and the need for clearer guidance after an ASD diagnosis.

**What is the implication of the main findings?**

- These findings highlight caregiver support, affordability, post-diagnosis guidance, and service coordination as promising priorities for family-centred ASD service development and prospective evaluation. Further research can identify which approaches most effectively strengthen maternal wellbeing.

**Abstract:**

Background: Mothers of children with autism spectrum disorder (ASD) play a central role in coordinating multidisciplinary interventions. This study examined quality of life (QoL) among Saudi mothers of children with ASD and its associations with caregiver burden, psychological distress, financial experiences, and perceived clarity of post-diagnosis service pathways. Methods: This cross-sectional quantitative survey included an exploratory free-text component. Of 109 survey submissions, 106 contained completed core survey data. Two brief open-ended questions were presented to 60 participants; 53 provided a post-diagnosis challenge response and 46 provided a general additional comment. These responses were reviewed descriptively as supplementary contextual observations. QoL was assessed using an adapted 22-item Arabic questionnaire derived from the Arabic WHOQOL-BREF source instrument and was analysed as an adapted QoL composite rather than as standard WHOQOL-BREF domain scores. Hierarchical linear regression used a fixed complete-case sample, and regression diagnostics and sensitivity analyses were conducted. Results: Ninety-nine mothers had sufficient valid responses to calculate the adapted QoL composite (mean = 50.65, SD = 17.32, on a 0–100 metric). The hierarchical regression included 82 participants and explained 59.3% of the variance in adapted QoL (R^2^ = 0.593; adjusted R^2^ = 0.536). Higher caregiver burden was associated with lower adapted QoL (B = −12.94, *p* < 0.001), whereas higher household income (B = 3.19, *p* = 0.040) and greater pathway clarity (B = 6.92, *p* = 0.005) were associated with higher adapted QoL. Psychological distress and perceived financial burden were associated with QoL in bivariate analyses but did not show statistically significant unique associations in the multivariable model. The free-text observations referred to caregiving demands, emotional adjustment, service-navigation uncertainty, and therapy affordability; they were not treated as formal qualitative findings. Conclusions: In this cross-sectional Saudi sample, caregiver burden emerged as the factor most strongly associated with lower adapted maternal QoL, whereas higher household income and clearer post-diagnosis service pathways were associated with better QoL. These findings highlight the importance of family-centred approaches that attend to caregiving demands, affordability, and access to clear post-diagnosis guidance. Prospective research using validated or fully documented measures should examine how caregiver and service-related factors can be addressed to strengthen maternal wellbeing over time.

## 1. Introduction

Autism spectrum disorder (ASD) is a neurodevelopmental condition characterized by persistent challenges in social communication and restricted or repetitive behaviors, often accompanied by language delays and sensory, behavioral, and adaptive difficulties. Children with ASD typically require long-term, multidisciplinary intervention, including speech–language pathology, occupational therapy, psychological services, and educational support. Consequently, caregiving demands are substantial and often extend well beyond routine parenting responsibilities.

A growing body of international literature has demonstrated that caregivers of children with ASD, particularly mothers, experience significantly reduced quality of life (QoL) compared with caregivers of typically developing children. Reductions have been reported across physical, psychological, social, and environmental domains of QoL and are frequently associated with caregiver burden, emotional distress, and chronic stress [1,2]. The World Health Organization Quality of Life instrument (WHOQOL-BREF) is a widely used multidimensional QoL instrument, and its Arabic source version has demonstrated acceptable psychometric properties in an Arab general-population sample [3].

In Saudi Arabia, the need for structured diagnostic and intervention services for children with ASD has received increasing attention. Available evidence indicates variation in service use and access, while parents have reported concerns regarding service accessibility, information and support, therapy costs, availability of specialised centres, and access to qualified providers [4,5]. Mothers are typically the primary caregivers and are more directly involved in daily care coordination, therapy attendance, and educational decision-making, which places them at higher risk of psychological and functional burden compared to other family members. Within the Saudi Arabian context, several studies have reported moderate to low quality of life among parents and caregivers of children with ASD, with mothers often reporting greater psychological strain than fathers [6,7]. While these studies provide important insights into caregiver wellbeing, most have focused on descriptive outcomes or caregiver stress without systematically examining system-level contributors to reduced quality of life.

Beyond individual and child-related factors, international research increasingly highlights the role of healthcare system structure in shaping caregiver experiences. Parents of children with ASD frequently report difficulties accessing appropriate services due to fragmented care systems, unclear referral processes, long waiting lists, and limited coordination across disciplines [8]. Following diagnosis, many families describe uncertainty regarding next steps, lack of guidance on prioritizing services, and the need to independently navigate complex health, rehabilitation, and educational systems [9]. Qualitative and systematic reviews consistently identify service navigation challenges as a major source of caregiver stress, particularly in settings where coordinated post-diagnosis pathways are not clearly established [10,11].

Financial burden represents another critical yet under-examined factor affecting families of children with ASD. Studies from multiple countries show that ASD-related services are frequently associated with high out-of-pocket costs, especially when services are delivered through private providers or when public services are limited in availability [12,13]. In Saudi Arabia, the healthcare landscape for ASD services is characterized by a mixed public–private system, with significant variation in service availability, coordination, and funding mechanisms across regions [4,5]. Many families rely on private services or out-of-pocket payments for specialized interventions.

Caregiver burden and financial burden are related but conceptually distinct dimensions of the caregiving context. Caregiver burden refers to the subjective emotional, functional, and time-related strain associated with providing ongoing care, whereas financial burden refers to perceived direct and indirect economic strain arising from ASD-related services and related household costs. Both may co-occur and may share variance; nevertheless, they may represent different potential targets for family support and service policy. In the present study, they were included in the analytical model to examine their unique associations with maternal QoL after accounting for the other variables in the model, rather than to assume that they are unrelated or that either has a causal effect on QoL [12,13,14].

Although caregiver burden and emotional distress among parents of children with ASD have been widely documented [1,2,6,7], fewer studies in Saudi Arabia have examined these caregiver factors alongside financial experiences and mothers’ perceptions of the clarity of post-diagnosis service pathways [6,7]. The contextual contribution of the present study is to consider caregiver burden and psychological distress together with perceived financial experiences and pathway clarity within a single Saudi-context analytical framework. Guided by the stress-process perspective [15], the study distinguished background characteristics, caregiver-level stress indicators, and contextual service-related experiences. Maternal and child characteristics were treated as background correlates; caregiver burden and psychological distress as proximal caregiver-level stress indicators; and perceived financial experiences, pathway clarity, and service source as contextual factors relevant to caregivers’ service experiences. This framework informed the analytical ordering of variables but did not assume temporal ordering, causal effects, or untested mediation pathways. Accordingly, this cross-sectional study aimed to assess overall maternal QoL among Saudi mothers of children with ASD and to examine its associations with caregiver burden, psychological distress, financial experiences, and perceived clarity of post-diagnosis service pathways. Two brief open-ended questions provided supplementary exploratory context regarding post-diagnosis challenges and general additional comments; they were interpreted as exploratory contextual observations rather than as a formal qualitative dataset. We hypothesised that greater caregiver burden and psychological distress would be associated with lower QoL.

## 2. Materials and Methods

### 2.1. Study Design

This study used a cross-sectional quantitative survey design with an exploratory free-text component. Two brief open-ended questions, included in the original IRB-approved survey instrument, elicited mothers’ descriptions of their most significant post diagnosis challenge and any additional comments. Owing to an inadvertent omission from the initial online survey configuration, these questions were activated after data collection had commenced and were presented to 60 participants. The responses were reviewed descriptively to provide supplementary context for caregiver-reported challenges and service-navigation concerns. Given their brief survey format, they were interpreted as exploratory contextual observations rather than as a formal qualitative dataset.

### 2.2. Participants and Setting

Participants were recruited through two pathways: clinical recruitment (mothers of children receiving services at King Abdullah bin Abdulaziz University Hospital and affiliated clinics) and community recruitment (via social media platforms and ASD parent support networks across Saudi Arabia). As the number of individuals approached or reached was not recorded consistently across the clinical and community recruitment channels, a recruitment response rate was not calculated.

Inclusion criteria required participants to be mothers aged 18 years or older, residents of Saudi Arabia, and the primary caregiver of a child aged 2–18 years with a caregiver-reported ASD diagnosis made by a qualified healthcare professional. Caregivers of children without a confirmed diagnosis, non-residents, and those with incomplete survey responses were excluded. Of 109 submitted records, 106 contained completed core survey data and were retained for descriptive analyses. A formal power analysis (G*Power 3.1) [16], conducted during study planning, indicated that a hierarchical multiple regression with up to seven regression variables, assuming a medium effect size (f^2^ = 0.15), alpha = 0.05, and power = 0.80, would require a minimum sample of 103 participants. The hierarchical regression included ten regression variables and a fixed complete-case sample of 82 participants; therefore, the original planning calculation should not be interpreted as confirmation of adequate power for this model, and the multivariable findings require replication in larger samples.

### 2.3. Measures

Data were collected using an online Arabic survey comprising established instruments or instrument-derived items and study-specific modules:

- Quality of Life: Quality of life was assessed using an Arabic questionnaire adapted from the World Health Organization Quality of Life–Brief instrument (WHOQOL-BREF). The Arabic WHOQOL-BREF source instrument has previously demonstrated acceptable psychometric properties in an Arab general-population sample [3]. The survey administered in the present study did not retain the full standard 26-item WHOQOL-BREF structure. Accordingly, the questionnaire was analysed as an adapted 22-item Arabic QoL composite rather than as a standard WHOQOL-BREF total or domain score. All item responses were directionally coded so that higher values represented better self-reported QoL; the pain/discomfort item and the two negatively worded frequency items were reverse scored. The participant-level composite was calculated as the mean of valid item scores and transformed to a 0–100 metric for respondents with at least 18 valid responses out of 22. Multi-selected or non-scale free-text entries to single-select QoL items were treated as unavailable for that item. A sensitivity analysis excluded the work-capacity item because three respondents entered non-scale employment/free-text responses.
- Child Characteristics: Mothers reported their child’s age, sex, age at ASD diagnosis, level of independence, and any co-occurring conditions from a checklist that allowed multiple selections. The checklist included intellectual disability, speech and language disorder or speech delay, attention-deficit/hyperactivity disorder (ADHD), anxiety disorder, seizure disorder, other conditions, and no reported co-occurring condition. These data were caregiver reports collected through the anonymous survey; clinical records, diagnostic reports, and standardised assessments were not reviewed to verify individual co-occurring conditions.
- Caregiver Burden: The Zarit Burden Interview short form (ZBI-6) [17] measured subjective caregiver burden. The six-item mean score was used, with higher scores indicating greater caregiver burden. Internal consistency in the present analytic dataset was Cronbach’s α = 0.662.
- Psychological Distress: Nine administered items from the Depression Anxiety Stress Scales (DASS-9) were summed to provide a total psychological-distress score [18], with higher scores indicating greater distress. Internal consistency in the present analytic dataset was Cronbach’s α = 0.819.
- Perceived Financial Burden: A three-item study-specific perceived-financial-burden score assessed (1) perceived financial pressure associated with ASD-related services, (2) the perceived impact of these costs on other household expenses, and (3) whether services had been reduced or discontinued because of cost. The first two items used ordered five-category response scales, coded from 1 to 5; the cost-related service-reduction item was coded No = 1 and Yes = 5. Higher scores indicated greater perceived financial burden, and the composite was calculated as the mean of the three coded items. Internal consistency was Cronbach’s α = 0.635 (complete-item *n* = 106). Monthly out-of-pocket expenditure and the estimated proportion of household income spent on ASD-related costs were treated as descriptive financial indicators and were not included in this composite. As this was a study-specific measure, its psychometric properties beyond internal consistency were not established, and its associations with QoL were interpreted as exploratory.
- Service Source: Service source refers to the respondent-reported usual source of the child’s ASD-related services and was categorized as public-only, mixed public/private, or private-only. In the regression model, mixed and private-only service-source indicators were entered, with public-only service use as the reference category.
- Post-Diagnosis Service Pathway Clarity: A seven-item study-specific module assessed guidance after diagnosis, referral timing, coordination across services, education-related guidance, difficulty navigating services independently, satisfaction with navigation, and navigation burden. Items were directionally aligned so that higher values represented greater pathway clarity or lower navigation burden. Each item was standardized before averaging because the response formats differed. A pathway-clarity score was calculated for participants with at least six valid items; “not sure” responses to the coordination item were treated as unavailable for that item. Internal consistency was Cronbach’s α = 0.756 among the 36 participants with complete responses to all seven items; a pathway-clarity score was available for 92 participants. This study-specific score was not subjected to formal content-validation or dimensionality testing in the present study; therefore, its psychometric properties require further evaluation and its associations with QoL are interpreted as exploratory. The item content, response coding, and score-construction procedures for the study-specific perceived-financial-burden and pathway-clarity measures are provided in Supplementary Table S1.
- Exploratory Free-Text Data: Two open-ended survey items invited mothers to describe the most significant challenge they had faced after their child’s ASD diagnosis and to provide any additional comments. These items were included to provide brief contextual information and were analysed as supplementary exploratory data; they were not designed to provide the depth of a formal qualitative study.

Caregiver burden and perceived financial burden were treated as related but nonidentical dimensions of the caregiving context. Caregiver burden captured subjective emotional, functional, and time-related strain associated with the caregiving role, whereas perceived financial burden captured economic strain related to ASD services and associated household costs [12,13,17]. Both variables were included in the multivariable analysis to examine their unique statistical associations with maternal QoL conditional on the other model variables; their inclusion did not assume that the constructs were unrelated or that either had a causal effect on QoL.

### 2.4. Data Collection and Ethical Considerations

Ethical approval was obtained from the Institutional Review Board at King Abdullah bin Abdulaziz University Hospital (IRB Log number: 26-0007). The study complied with the Declaration of Helsinki and Saudi Arabian research ethics guidelines. Electronic informed consent was obtained from all participants prior to survey completion. Surveys were anonymous, and data were stored on a secure, encrypted server.

### 2.5. Data Analysis

Quantitative analyses were conducted using Python version 3.12 (Python Software Foundation, Wilmington, DE, USA), with the pandas, SciPy, and statsmodels packages. Descriptive statistics were generated for all variables. Quality of life was operationalized as an adapted 22-item Arabic QoL composite because the administered survey did not retain the full standard 26-item WHOQOL-BREF structure. All items were directionally coded so that higher values indicated better self-reported QoL. The pain/discomfort item and the two negatively worded frequency items were reverse-scored. Participant-level QoL scores were calculated as the mean of valid item scores, transformed to a 0–100 metric, for participants with at least 18 valid item responses. Responses that were multi-selected or entered as non-scale free text for a single-select QoL item were treated as unavailable for that item. A sensitivity analysis excluded the work-capacity item because three respondents entered non-scale employment/free-text responses.

Of 109 submitted records, 106 contained completed core survey data. Closed survey fields were complete in these 106 records. However, the adapted QoL composite and selected derived predictors could be unavailable after direction coding, treatment of non-scale responses, and application of prespecified scoring rules. Variable-level completion and missingness, including the denominator for the exploratory free-text prompts, are reported in Supplementary Table S2. No values were imputed. Descriptive summaries report the observed denominator for each variable. Pearson correlations used pairwise complete observations, and the sample size for each correlation is reported alongside the corresponding correlation coefficient. The hierarchical linear regression used a fixed complete-case sample for 82 participants with valid values for the adapted QoL composite and all ten regression variables across all four model steps.

The hierarchical regression model was specified using the stress-process framework [15]. Maternal age, education, household income, and child independence were entered at Step 1 as background sociodemographic and child-related characteristics that provide context for caregiver experiences. Caregiver burden and psychological distress were entered at Step 2 as proximal caregiver-level stress indicators. Perceived financial burden was entered at Step 3 as a contextual economic stressor. Pathway clarity and service source were entered at Step 4 as service-related experiences that may be associated with QoL beyond the preceding background, caregiver-level, and financial variables. Service source refers to the respondent-reported usual source of the child’s ASD-related services and was categorized as public-only, mixed public/private, or private-only. In the regression model, mixed and private-only service-source indicators were entered, with public-only service use as the reference category. This block order was used to organise the analysis according to the conceptual framework; it does not establish temporal sequence, causal direction, or mediation.

Regression assumptions were assessed for the final model. Linearity and homoscedasticity were evaluated by visual inspection of residual-versus-fitted plots, supplemented by the Breusch–Pagan test. Residual normality was assessed by Q–Q plot review and the Shapiro–Wilk test. Independence of residuals was assessed descriptively using the Durbin–Watson statistic. Multicollinearity was evaluated using tolerance values and variance-inflation factors (VIFs). Influence was assessed using Cook’s distance and leverage. HC3 robust-standard-error and influence-sensitivity analyses were conducted to examine the robustness of the final estimates. Regression coefficients are reported with 95% confidence intervals.

Two open-ended survey items provided supplementary contextual information on post-diagnosis challenges and general additional comments. Responses were reviewed descriptively rather than analysed as a formal qualitative dataset. Of the 60 participants who were presented with the questions, 53 provided a response to the post-diagnosis challenge question; each response was assigned one primary descriptive category for reporting. Forty-six participants provided a general additional comment, which was reviewed as non-numerical contextual information.

## 3. Results

### 3.1. Participant Characteristics

A total of 109 survey submissions were received. Of these, 106 contained completed core survey data and were retained for the descriptive analyses. Among the 106 core-complete records, 99 participants had sufficient valid responses to calculate the adapted QoL composite; variable-level completion and missingness are reported in Supplementary Table S2. Most mothers of children with ASD were aged 36–55 years (64.1%), held a bachelor’s degree (62.3%), and were homemakers (58.5%). The majority of children were male (70.8%), aged 6–10 years (55.7%), and received their ASD diagnosis between 2 and 3 years of age (49.1%). Regarding service source, 50.0% used public services exclusively, 30.2% used a combination of public and private, and 19.8% relied solely on private services. Caregiver-reported speech and language disorder was the most frequently reported co-occurring condition (87/106, 82.1%), followed by caregiver-reported ADHD (71/106, 67.0%). Detailed participant characteristics are presented in Table 1. City of residence was reported by 78 of the 106 core-complete participants; 28 city responses were unavailable, and the free-text entries were not recoded into geographic regions.

### 3.2. Adapted Arabic QoL Composite

Among the 106 completed core survey records, 99 participants had sufficient valid responses to calculate the adapted 22-item Arabic QoL composite. The mean adapted QoL score was 50.65 (SD = 17.32) on a 0–100 metric. Internal consistency across the 22 administered items was Cronbach’s α = 0.754 among the 13 participants with complete responses to all 22 items. However, this estimate was based on only 13 complete-item responses and should therefore be interpreted cautiously. In a sensitivity analysis excluding the work-capacity item, the mean score was 50.87 (SD = 17.28) and Cronbach’s α was 0.751 among the 13 complete-item cases. These results are interpreted as descriptive properties of an adapted composite rather than as standard WHOQOL-BREF total or domain scores.

### 3.3. Bivariate Associations

Bivariate associations with the adapted 22-item Arabic QoL composite are shown in Table 2. Higher caregiver burden, psychological distress, and perceived financial burden were associated with lower adapted QoL, whereas greater child independence and pathway clarity were associated with higher adapted QoL.Maternal age, maternal education, and household income were not significantly associated with adapted QoL in bivariate analyses.

### 3.4. Hierarchical Regression and Diagnostics

A hierarchical linear regression was estimated using a fixed complete-case sample of 82 participants (Table 3). A descriptive comparison of the 82 retained participants and the 24 core-complete records not retained because of unavailable outcome or regression-variable values found that the retained participants had higher child-independence scores (mean 2.04 vs. 1.67; Welch t = 2.405, *p* = 0.019). No statistically significant observed differences were identified for maternal age, maternal education, household income, caregiver burden, psychological distress, perceived financial burden, pathway clarity, or service-source distribution (all *p* > 0.05). Demographic and child covariates did not explain a statistically significant proportion of adapted QoL variance at Step 1 (R^2^ = 0.088, *p* = 0.125). Adding caregiver burden and psychological distress at Step 2 explained an additional 42.9% of variance (ΔR^2^ = 0.429, *p* < 0.001). Adding perceived financial burden at Step 3 did not increase explained variance (ΔR^2^ = 0.000, *p* = 0.997). Adding pathway clarity and service source at Step 4 explained a further 7.5% of variance (ΔR^2^ = 0.075, *p* = 0.007). The multivariable model explained 59.3% of the variance in adapted QoL (R^2^ = 0.593; adjusted R^2^ = 0.536; F(10, 71) = 10.346, *p* < 0.001).

**Figure 1:**
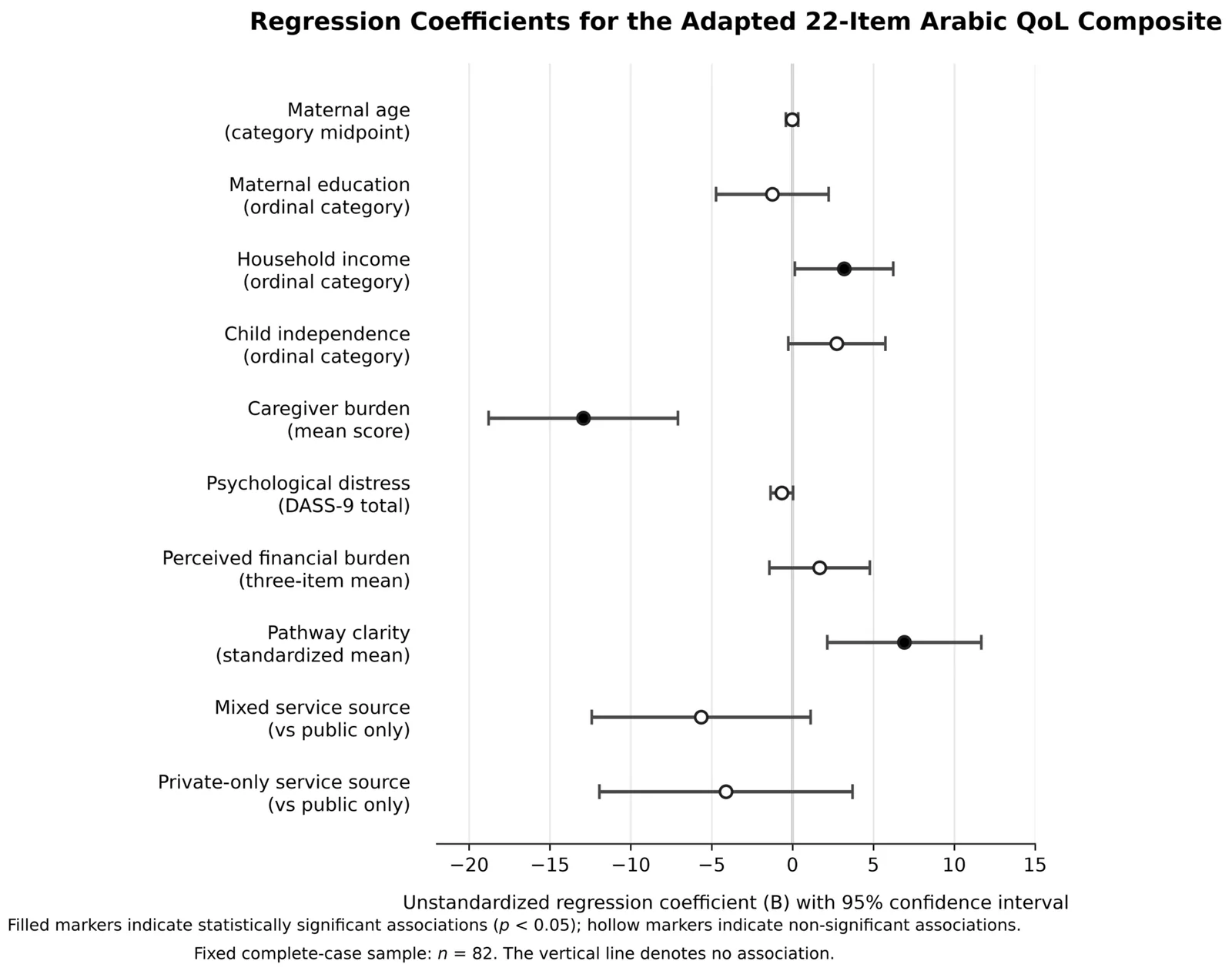
Regression coefficients for the adapted 22-item Arabic QoL composite. Points show unstandardized regression coefficients and horizontal bars show 95% confidence intervals (n = 82). Filled markers indicate statistically significant associations; hollow markers indicate non-significant associations.

Higher caregiver burden was associated with lower adapted QoL (B = −12.94, 95% CI [−18.80, −7.08], *p* < 0.001), whereas higher household income (B = 3.19, 95% CI [0.15, 6.24], *p* = 0.040) and greater pathway clarity (B = 6.92, 95% CI [2.16, 11.68], *p* = 0.005) were associated with higher adapted QoL. Psychological distress, perceived financial burden, child independence, service source, maternal age, and maternal education did not show statistically significant unique associations in the multivariable model. Regression coefficients and 95% confidence intervals are shown in Figure 1.

Diagnostic assessment did not identify a material violation of linear-model assumptions. Residual-versus-fitted and Q–Q plots were reviewed; residuals were consistent with approximate normality (Shapiro–Wilk W = 0.982, *p* = 0.322), and the Breusch–Pagan test did not indicate heteroscedasticity (*p* = 0.700). The Durbin–Watson statistic was 1.727. Multicollinearity was not problematic (maximum VIF = 2.233; minimum tolerance = 0.448). The VIFs for caregiver burden and psychological distress were 2.233 and 1.683, respectively. Four observations exceeded the heuristic Cook’s-distance threshold. HC3 robust-standard-error sensitivity retained the statistical significance of caregiver burden, household income, and pathway clarity. In influence-sensitivity analyses, the caregiver-burden and pathway-clarity associations retained their directions, whereas selected estimates for regression variables that did not show statistically significant unique associations were sensitive to influential observations and should be interpreted cautiously.

### 3.5. Exploratory Free-Text Responses

Of the 60 participants who were presented with the two open-ended questions, 53 mothers provided a response to the question on the most significant challenge experienced after their child’s ASD diagnosis, and 46 provided a general additional comment. The responses were treated as supplementary exploratory observations rather than as formal qualitative findings. Each of the 53 challenge responses was assigned one primary descriptive category. The most frequently represented categories were communication and behavioural demands (13/53, 24.5%), emotional shock and psychological adjustment (12/53, 22.6%), navigating an unfamiliar service system (10/53, 18.9%), social stigma and community barriers (9/53, 17.0%), and fear and uncertainty about the future (8/53, 15.1%). One respondent reported no post-diagnosis challenge (1/53, 1.9%). These percentages represent the proportion of the 53 challenge responses assigned to each primary descriptive category; they are not prevalence estimates for the full study sample. The general additional comments included references to service access, therapy affordability, caregiver support, and the need for clearer guidance after diagnosis. As this item was a general additional-comments prompt rather than a structured support-needs question, it is not reported as a separate numerical support-needs analysis. These exploratory free-text observations provide limited context for caregiver-reported challenges and service-related concerns. They are not presented as formal qualitative themes or as evidence that confirms the quantitative findings.

## 4. Discussion

This cross-sectional quantitative survey with an exploratory free-text component examined maternal QoL in relation to caregiver burden, psychological distress, financial experiences, and post-diagnosis service-pathway experiences among Saudi mothers of children with ASD. The study considered caregiver and service-related factors together within a Saudi-context framework. The free-text observations provide supplementary descriptive context for caregiver-reported concerns and do not constitute a formal qualitative analysis. Higher caregiver burden was associated with lower adapted QoL, whereas higher household income and greater pathway clarity were associated with higher adapted QoL in the multivariable model. Psychological distress and perceived financial burden were associated with lower adapted QoL in bivariate analyses but did not show statistically significant unique associations in the multivariable model. The regression model explained 59.3% of the variance in adapted QoL; these findings should be interpreted as cross-sectional associations rather than evidence of temporal or causal relationships.

QoL was assessed using an adapted 22-item Arabic questionnaire derived from the Arabic WHOQOL-BREF source instrument [3]. As the survey did not retain the standard 26-item structure, the findings are reported for an adapted Arabic QoL composite rather than as standard WHOQOL-BREF total or domain scores. The reliability and validity evidence for the complete Arabic WHOQOL-BREF does not automatically apply to the adapted item set used here. Future research should evaluate the psychometric performance of the adapted measure in Saudi caregivers and, where domain-level inference is required, use the complete validated Arabic WHOQOL-BREF or another appropriately validated measure.

The stress-process framework provides a useful conceptual lens for organising caregiver-level and contextual factors [15]. Financial experiences and post-diagnosis pathway concerns may be relevant contextual stressors; however, the present cross-sectional data did not test temporal ordering, mediation, or other mechanisms linking these factors with caregiver burden, psychological distress, and QoL. Longitudinal studies with prespecified mediation analyses are needed to evaluate such pathways.

The high caregiver-reported prevalence of speech and language disorder among children in this sample (82.1%) warrants specific attention in the context of maternal QoL. Speech–language pathology services are among the most frequently accessed services for young children with autism, as parents consistently identify language and social communication skills as treatment priorities [19]. Communication difficulties not only impede the child’s social participation and learning but also place considerable daily demands on caregivers, who must serve as communication facilitators and advocates across home, therapy, and educational settings [20]. Future research should examine the associations of speech–language therapy access, cost, and coordination with caregiver wellbeing using prospectively specified measures.

Perceived financial burden was associated with lower adapted QoL in bivariate analyses but did not show a statistically significant unique association in the multivariable model. The three-item perceived-financial-burden measure was study-specific, and the cross-sectional design does not establish temporal or causal relationships among financial experiences, caregiver burden, psychological distress, and QoL. Financial experiences remain an important service-related aspect of caregiving and warrant prospective investigation using prespecified, validated measures that distinguish direct costs, household resources, insurance coverage, and service availability.

Greater pathway clarity was associated with higher adapted QoL in both bivariate and multivariable analyses. Pathway clarity was assessed using a study-specific measure that has not undergone full validation; therefore, this exploratory finding requires confirmation in studies using a validated measure. Post-diagnosis service navigation remains an important caregiver-reported area for further study. The general additional comments included references to service access, therapy affordability, caregiver support, and the need for clearer guidance after diagnosis. These brief observations identify caregiver-reported concerns for future study but do not provide formal evidence of convergence, divergence, or mechanisms linking financial experiences or pathway clarity to QoL. They should not be used to explain the multivariable results or to infer that a particular navigation intervention would improve maternal QoL. Prospective and implementation research is needed to examine whether, and under what conditions, clearer post-diagnosis guidance and coordination improve caregiver experiences.

### 4.1. Implications for Policy and Practice

The observed associations identify priorities for further family-centred ASD service research. Caregiver burden was associated with lower adapted maternal QoL, whereas pathway clarity was associated with higher adapted QoL in the multivariable model. These findings support prospective evaluation of approaches that address caregiver burden and improve the clarity, coordination, and accessibility of services after diagnosis. They do not establish that psychological support, respite care, service coordination, financial assistance, or digital-navigation programmes improve maternal QoL. Perceived financial burden was associated with lower adapted QoL in bivariate analyses but did not show a statistically significant unique association in the multivariable model. Financial experiences remain an important service-related area for prospective research using prespecified measures. Policymakers and researchers may examine how the availability, affordability, and funding of ASD services are associated with family experiences. The present cross-sectional study does not establish the effect of a particular funding model.

Post-diagnosis information, referral, and coordination approaches warrant prospective evaluation. A centralised digital navigation platform is one possible service-development approach that could be evaluated in Saudi Arabia. Such a platform could provide families with information about available services, including speech–language therapy, occupational therapy, psychological services, and inclusive educational placements, according to geographic location and child characteristics. Emerging international research has explored whether digital service-navigation tools may reduce the burden of navigating fragmented care systems and improve access to appropriate services, particularly for lower-resourced families [21]. However, this study did not assess a digital navigation platform and cannot establish its feasibility, acceptability, accessibility, effectiveness, cost-effectiveness, data-governance requirements, or whether it would improve maternal wellbeing in Saudi Arabia. Any future programme should be co-designed with caregivers and service providers and evaluated prospectively for feasibility, accessibility, acceptability, effectiveness, equity, data governance, and cost-effectiveness before wider implementation. These directions are consistent with international calls for coordinated, family-centred ASD service models [22,23] and may be particularly relevant in the Saudi context, where post-diagnosis family support warrants further study.

### 4.2. Limitations

Several limitations must be acknowledged. First, the QoL questionnaire administered in this study was adapted from the Arabic WHOQOL-BREF and did not retain the full standard 26-item structure. Although the Arabic source instrument has demonstrated acceptable psychometric properties [3], those properties cannot be assumed to apply unchanged to the adapted item set. The outcome used in this study should therefore be described as an adapted 22-item Arabic QoL composite, not as a standard WHOQOL-BREF total or validated domain score.

Second, the cross-sectional design precludes causal inference; the temporal relationships among caregiver burden, psychological distress, financial experiences, pathway clarity, and maternal QoL cannot be established. Caregiver burden and perceived financial burden may also share conceptual and statistical variance; although both were included to examine their unique associations with QoL, their overlap may influence the precision of individual estimates.

Third, child clinical characteristics, including co-occurring conditions, were based on caregiver report and did not include standardised ASD severity, adaptive-functioning, behavioural-difficulty, or comorbidity assessments; residual misclassification and confounding are therefore possible.

Fourth, the hierarchical regression used a fixed complete-case sample of 82 participants. Although this approach enabled consistent comparison of hierarchical blocks, it reduced the analytic sample and may have affected the precision and stability of estimates. Participants retained in the complete-case sample had higher child-independence scores than those not retained; no statistically significant observed differences were identified for the other compared characteristics. However, these comparisons do not establish the missing-data mechanism, and unmeasured differences between retained and non-retained records remain possible. No imputation was performed.

Fifth, four observations exceeded the heuristic Cook’s-distance threshold, and influence sensitivity analyses showed that some estimates were sensitive to these observations.

Sixth, the perceived-financial-burden and pathway-clarity measures were study-specific. Their internal consistency estimates do not establish content validity, construct validity, dimensionality, or measurement invariance. Their psychometric performance therefore requires further evaluation, and their associations with adapted QoL should be interpreted as exploratory.

Seventh, the sample was predominantly well educated and was recruited through online, tertiary-service, and parent-network pathways. These recruitment methods may have introduced selection bias by favouring mothers with digital access, connections to specialist services or parent networks, and willingness to participate. As individual-level recruitment source was not retained in the anonymous survey export, the study could not assess whether recruitment pathway was associated with caregiver burden, service use, pathway experiences, or QoL. City of residence was reported by 78 participants and was missing for 28; the free-text entries were not recoded into regions. The study therefore could not assess regional or rural–urban variation in caregiver experiences. These limitations may underrepresent mothers from lower-resource, rural, digitally disconnected, primary-care, or underserved settings. The findings are not necessarily generalisable to all mothers of children with ASD in Saudi Arabia or to populations in other countries with different cultural, healthcare, and service-delivery contexts. The use of self-reported measures for caregiver experiences, quality of life, and child clinical characteristics may have introduced recall or reporting bias. As these variables were collected from the same respondent in a single survey, common-method variance may also have influenced the observed associations.

Finally, the study focused exclusively on mothers, and the two brief open-ended survey questions did not provide the depth, probing, or procedural rigour of qualitative interviews or a dedicated qualitative study. The free-text observations were descriptive and hypothesis-generating rather than formal qualitative themes, prevalence estimates, or confirmatory evidence for the quantitative associations.

## 5. Conclusions

In this cross-sectional Saudi sample, caregiver burden emerged as the factor most strongly associated with lower adapted maternal QoL, whereas higher household income and clearer post-diagnosis service pathways were associated with better QoL. These findings highlight the importance of family-centred approaches that attend to caregiving demands, affordability, and access to clear post-diagnosis guidance. Prospective research using validated or fully documented measures should examine how caregiver and service-related factors can be addressed to strengthen maternal wellbeing over time.

## Figures and Tables

**Table 1:** Participant and Child Characteristics (*N* = 106).

Characteristic	n (%)
Maternal Age	
26–35 years	33 (31.1%)
36–55 years	68 (64.1%)
Other	5 (4.8%)
Maternal Education	
Secondary school	17 (16.0%)
Bachelor’s degree	66 (62.3%)
Other	23 (21.7%)
Employment Status	
Homemaker	62 (58.5%)
Employed full-time	37 (34.9%)
Other	7 (6.6%)
Marital Status	
Married	94 (88.7%)
Other	12 (11.3%)
Child Sex	
Male	75 (70.8%)
Female	31 (29.2%)
Child Age	
2–5 years	26 (24.5%)
6–10 years	59 (55.7%)
11–18 years	21 (19.8%)
Age at ASD Diagnosis	
2–3 years	52 (49.1%)
Other	54 (50.9%)
Service Source	
Public services only	53 (50.0%)
Mixed (public and private)	32 (30.2%)
Private services only	21 (19.8%)
Caregiver-Reported Co-Occurring Conditions (multiple responses allowed)	
Speech and language disorder	87 (82.1%)
ADHD	71 (67.0%)
Anxiety	21 (19.8%)
Intellectual disability	18 (17.0%)

Note. Co-occurring conditions were reported by mothers through the survey checklist and were not verified through clinical-record review, diagnostic reports, or standardised assessments. Percentages therefore describe caregiver-reported conditions in this sample and may not represent clinically confirmed prevalence.

**Table 2:** Bivariate Associations with the Adapted 22-Item Arabic QoL Composite.

Variable	n	Pearson r	*p*
Maternal age	99	0.051	0.613
Maternal education	96	−0.054	0.598
Household income	95	0.161	0.119
Child independence	99	0.227	0.024
Caregiver burden	99	−0.621	<0.001
Psychological distress	99	−0.542	<0.001
Perceived financial burden	99	−0.357	<0.001
Pathway clarity	86	0.456	<0.001

Note. Pairwise complete observations were used, so the denominator varies across correlations. The pathway-clarity score was available for 92 records; its bivariate analysis with the adapted QoL composite included 86 records.

**Table 3:** Hierarchical Regression of the Adapted 22-Item Arabic QoL Composite (n = 82).

Regression Variable	B	SE	Standardized β	95% CI for B	t	*p*
Intercept	70.882	12.442	—	[46.073, 95.690]	5.697	<0.001
Maternal age (years; category midpoint)	−0.023	0.192	−0.010	[−0.407, 0.360]	−0.122	0.903
Maternal education (ordinal category)	−1.242	1.746	−0.062	[−4.723, 2.239]	−0.711	0.479
Household income (ordinal category)	3.192	1.526	0.202	[0.148, 6.235]	2.091	0.040
Child independence (ordinal category)	2.742	1.506	0.145	[−0.260, 5.745]	1.821	0.073
Caregiver burden (mean score)	−12.938	2.938	−0.498	[−18.796, −7.080]	−4.404	<0.001
Psychological distress (DASS-9 total)	−0.663	0.351	−0.186	[−1.362, 0.036]	−1.892	0.063
Perceived financial burden (three-item mean)	1.676	1.559	0.110	[−1.433, 4.784]	1.075	0.286
Pathway clarity (standardized mean)	6.918	2.388	0.279	[2.157, 11.679]	2.898	0.005
Mixed service source (vs. public only)	−5.648	3.397	−0.323	[−12.421, 1.125]	−1.663	0.101
Private-only service source (vs. public only)	−4.118	3.923	−0.236	[−11.940, 3.704]	−1.050	0.297

Model summary. Step 1 (maternal age, education, income, and child independence): R^2^ = 0.088, adjusted R^2^ = 0.041, F(4, 77) = 1.865, *p* = 0.125. Step 2 (caregiver burden and psychological distress): ΔR^2^ = 0.429, F change(2, 75) = 33.403, *p* < 0.001. Step 3 (perceived financial burden): ΔR^2^ = 0.000, F change(1, 74) = 0.000, *p* = 0.997. Step 4 (pathway clarity and service source): ΔR^2^ = 0.075, F change(3, 71) = 4.374, *p* = 0.007. Final model: R^2^ = 0.593, adjusted R^2^ = 0.536, F(10, 71) = 10.346, *p* < 0.001. Note. The outcome was the adapted 22-item Arabic QoL composite transformed to a 0–100 metric. The fixed complete-case sample was used for all steps. Public-only service source was the reference category. Service source refers to the respondent-reported usual source of the child’s ASD-related services. The final model included maternal age, maternal education, household income, child independence, caregiver burden, psychological distress, perceived financial burden, pathway clarity, and service-source indicators. Results are cross-sectional associations. An em dash (—) indicates that a standardised coefficient was not applicable to the intercept.

## Data Availability

The data presented in this study are available on request from the corresponding author. The data are not publicly available due to privacy and ethical restrictions.

## Supplementary Materials

The following supporting information can be downloaded at: https://www.mdpi.com/article/10.3390/healthcare14172847/s1, Table S1: Study-Specific Financial-Burden and Post-Diagnosis Pathway-Clarity Measures: Items, Coding, and Scoring; Table S2: Completion and Missingness in the Survey Export.

## Abbreviations

The following abbreviations are used in this manuscript:

ASD	Autism Spectrum Disorder
QoL	Quality of Life
WHOQOL-BREF	World Health Organization Quality of Life—Brief Version
ZBI-6	Zarit Burden Interview—Short Form (6-item)
DASS-9	Depression Anxiety Stress Scales—Short Form (9-item)
IRB	Institutional Review Board
SD	Standard Deviation
GCC	Gulf Cooperation Council
CRediT	Contributor Roles Taxonomy
