# Supplementary material for: Quality of Life Among Saudi Mothers of Children with Autism Spectrum Disorder: Associations with Caregiver Burden, Psychological Distress, Financial Experiences, and Post-Diagnosis Service Pathway Clarity
Source: Healthcare (Basel). 2026 Sep 4;14(17):2847. doi: 10.3390/healthcare14172847 (PMC13565271; doi:10.3390/healthcare14172847)
Supplement: Supplementary file 1 [file healthcare-14-02847-s001.zip › healthcare-4483716-supplementary.pdf]

Supplementary Table S1. Study-Specific Financial-Burden and Post-Diagnosis Pathway-Clarity Measures: Items, Coding, and Scoring

Measure	Item / construct	Response format and coding	Score construction
Perceived financial burden	Financial pressure associated with ASD-related services	Five ordered categories, coded 1 = no pressure to 5 = very severe pressure	Mean of the three coded items; higher score = greater perceived financial burden
Perceived financial burden	Perceived effect of ASD-related costs on other household expenses	Five ordered categories, coded 1 = no impact to 5 = very severe impact	Included in the three-item mean
Perceived financial burden	Reduction or discontinuation of ASD services because of cost	No = 1; Yes = 5	Included in the three-item mean
Pathway clarity	Guidance after diagnosis	Four ordered response categories, directionally coded 1, 2, 4, and 5; higher = clearer guidance	Standardized before averaging with the pathway items
Pathway clarity	Referral timing	Five ordered response categories, directionally coded 1–5; higher = more timely referral	Standardized before averaging with the pathway items
Pathway clarity	Coordination across services	Four ordered response categories, directionally coded 1, 2, 4, and 5; "not sure" treated as unavailable; higher = greater coordination	Standardized before averaging with the pathway items
Pathway clarity	Education-related guidance	Five ordered response categories, directionally coded 1–5; higher = clearer guidance	Standardized before averaging with the pathway items
Pathway clarity	Difficulty navigating services independently	Five ordered response categories, reverse-direction coded 5–1; higher = less difficulty	Standardized before averaging with the pathway items
Pathway clarity	Satisfaction with navigation	Five ordered satisfaction categories, coded 1–5; higher = greater satisfaction	Standardized before averaging with the pathway items
Pathway clarity	Navigation burden	Five ordered response categories, reverse-direction coded 5–1; higher = lower navigation burden	Standardized before averaging with the pathway items

Note. The perceived-financial-burden composite was available for 106 participants and had Cronbach's α = .635 among complete-item cases (n = 106). Monthly out-of-pocket expenditure and the estimated proportion of household income spent on ASD-related costs were reported descriptively and were not included in this composite. The pathway-clarity score was calculated when at least six of seven items were valid and was available for 92 participants. Cronbach's α = .756 among the 36 participants with complete responses to all seven pathway items. Both measures were study-specific and should be interpreted as exploratory; the reported internal-consistency estimates do not establish content validity, construct validity, or dimensionality.

Supplementary Table S2. Completion and Missingness in the Survey Export

The table reports aggregate completion information for the 106 core-complete survey records. It does not contain participant identifiers or free-text responses.

Variable group or field	Number of fields	Eligible records	Observed responses per field	Missing responses among eligible records	Missingness among eligible records	Role in study
Maternal socio-demographic variables	7	106	106	0	0.0%	Main quantitative data collection
Child and service characteristics	8	106	106	0	0.0%	Main quantitative data collection
Financial-experience questions	5	106	106	0	0.0%	Main quantitative data collection
Post-diagnosis pathway/navigation questions	7	106	106	0	0.0%	Main quantitative data collection
Adapted Arabic QoL item fields	22	106	106	0	0.0%	Main quantitative data collection
Caregiver-burden item fields	6	106	106	0	0.0%	Main quantitative data collection
Psychological-distress item fields	9	106	106	0	0.0%	Main quantitative data collection
City of residence	1	106	78	28	26.4%	Supplementary descriptive field
Post-diagnosis challenge free-text prompt	1	60	53	7	11.7%	Supplementary exploratory component
General additional-comment free-text prompt	1	60	46	14	23.3%	Supplementary exploratory component

Note. Closed survey fields included in the main quantitative data collection were complete for all 106 core-complete records. The two free-text questions were presented to 60 participants; the response counts reported above therefore use 60 as the denominator. The table describes raw survey-field completion. Availability for a derived analysis can differ after direction coding, treatment of non-scale entries, and complete-case selection; the relevant denominators are reported with each analysis.

https://doi.org/10.3390/xxxxx
